# Effects of Water-Nitrogen Management on the Growth and Nitrogen Uptake and Utilization of Intercropped Alfalfa

**DOI:** 10.3390/plants14162572

**Published:** 2025-08-19

**Authors:** Huile Lv, Yuanbo Jiang, Guangping Qi, Minhua Yin, Yanxia Kang, Yanlin Ma, Yayu Wang, Feng Xiao, Jianqing Peng, Haiyan Li, Chongqin Luo, Junxian Chen, Yanbiao Wang, Mingzhu Wang

**Affiliations:** 1 College of Water Conservancy and Hydropower Engineering, Gansu Agricultural University, Lanzhou 730070, China; 1073323020364@st.gsau.edu.cn (H.L.); 1073323010121@st.gsau.edu.cn (Y.J.); yinmh@gsau.edu.cn (M.Y.); yanxiakang@gsau.edu.cn (Y.K.); mayl@gsau.edu.cn (Y.M.); xiaof@gsau.edu.cn (F.X.); 107332201100@st.gsau.edu.cn (H.L.); 1073324120809@st.gsau.edu.cn (C.L.); 1073323020360@st.gsau.edu.cn (J.C.); 1073323020378@st.gsau.edu.cn (Y.W.); 1073324020379@st.gsau.edu.cn (M.W.); 2 Gansu Province Jingtai River Electric Power Irrigation Administration Bureau Irrigation Experiment Station, Baiyin 730400, China; 18152187772@163.com

**Keywords:** intercropping system, stem-to-leaf ratio, biomass allocation, nitrogen allocation, yield

## Abstract

Agroforestry is an ecological agricultural model that promotes the coordinated development of agriculture and animal husbandry. Exploring appropriate water and nitrogen management strategies for forage grasses in agroforestry systems is of great significance for improving productivity. This study aims to investigate the effects of different water and nitrogen management practices on the growth, nitrogen uptake, and utilization efficiency of intercropped alfalfa in a goji berry-alfalfa system. It is assumed that moderate water deficiency combined with appropriate nitrogen fertilizer can optimize the growth of alfalfa in the intercropping of wolfberry and alfalfa. This study was based on a 2-year (2021 and 2022) field trial, focusing on alfalfa in a goji berry||alfalfa system. Four irrigation levels [full irrigation (W0, 75–85% θ_fc_), mild water deficit (W1, 65–75% θ_fc_), moderate water deficit (W2, 55–65% θ_fc_), and severe water deficit (W3, 45–55% θ_fc_)] and four nitrogen application levels [no nitrogen (N0, 0 kg·hm^−2^), low nitrogen (N1, 150 kg·hm^−2^), medium nitrogen (N2, 300 kg·hm^−2^), and high nitrogen (N3, 450 kg·hm^−2^)] were set up to systematically analyze the effects of water and nitrogen regulation on biomass allocation, nitrogen translocation, hay yield, and nitrogen use efficiency of alfalfa. The results showed that (1) irrigation and nitrogen application levels significantly affected the stem-to-leaf and root-to-shoot ratios of alfalfa (*p* < 0.01). The smallest stem-to-leaf ratio (0.758) was observed under W1N2, while the smallest root-to-shoot ratio (0.595) was observed under W0N2. (2) Irrigation and nitrogen application levels significantly affected nitrogen accumulation and nitrogen translocation in alfalfa (*p* < 0.05). The maximum nitrogen accumulation was observed under W0N2, which was 43.39% higher than that under W0N0. The maximum nitrogen translocation was observed under W1N2, which was 15.1% and 33.4% higher on average than that under W0N0 and W3N0, respectively. (3) Irrigation and nitrogen application had highly significant effects on alfalfa hay yield (*p* < 0.01). The highest hay yield (8325 kg·hm^−2^ and 12,872 kg·hm^−2^) was achieved under W0N2. The nitrogen productivity of alfalfa increased with increasing water deficit and initially increased, then decreased with increasing nitrogen application. The nitrogen use efficiency of alfalfa followed the order N2 > N1 > N3 and W1 > W0 > W2 > W3, with the highest value of 9.26 under W1N2. Based on the comprehensive evaluation of alfalfa in agroforestry systems under water and nitrogen regulation using the entropy weight-TOPSIS method, mild water deficit combined with medium nitrogen application (W1N2) can optimize the stem-to-leaf ratio, root-to-shoot ratio, and nitrogen use efficiency of alfalfa without significantly reducing yield and nitrogen production efficiency. This water-nitrogen combination is suitable for use in goji berry||alfalfa systems in the Yellow River irrigation area of Gansu Province and similar ecological zones.

## 1. Introduction

With the continuous growth of the global population, the livestock industry is experiencing rapid development, and the demand for high-quality forage is increasing [1]. Alfalfa (*Medicago sativa* L.), a widely cultivated high-quality leguminous forage, has an annual planting area exceeding 33 million hectares worldwide and is acclaimed as the “King of Forages.” Owing to its high yield, palatability, nutritional value, and multiple ecological adaptations, such as soil improvement, windbreak, and sand fixation, alfalfa plays an important role in the intensive development of ruminant livestock in many countries and regions globally [2]. Nevertheless, the 2025 United Nations World Water Development Report (WWDR) reveals that 25 nations, which account for a quarter of the world’s population, are grappling with “extremely high” water stress levels. Moreover, roughly half of the global population experiences significant water scarcity during some portion of the year. Arridina et al. [3] indicated in their study that the global arable land area has increased by approximately 13% since 1961. However, due to the doubling of the world’s population, only half of the land previously available is now utilized for food production [4]. Issues such as water scarcity and the reduction of arable land have further strained forage supply. In some arid and semi-arid regions, high inputs of water and fertilizer are often employed to increase the yield of alfalfa, which not only leads to the waste of water resources but also causes a series of problems, such as environmental pollution and reduced fertilizer efficiency [5].

Rational cropping patterns and field management practices are crucial for improving material cycling and energy flow in agricultural ecosystems, as well as for mitigating soil erosion and environmental pollution [6]. Therefore, the exploration of efficient and environmentally friendly cultivation methods to address the shortage of forage and alleviate environmental burdens has become an urgent issue worldwide. Agroforestry, an ecological agricultural practice [7], involves planting herbaceous plants between rows of woody plants. This not only fully leverages the ecological protective functions of trees but also provides forage for animal husbandry, thereby achieving synergistic improvement in economic benefits and environmental protection. In arid and semi-arid regions, agroforestry plays an extremely important role in the sustainable development of agriculture and animal husbandry. Poloko et al. [8] found that intercropping is an important cropping system, which has advantages in weed management, soil fertility conservation, and increasing crop yields during climate change. Agnese et al. [9] assessed the effects of traditional soil management versus intercropping alfalfa between rows in vineyards on the physicochemical properties of soil on hilly vineyard lands in Tuscany and found that, compared with conventional tillage, alfalfa intercropping influenced the emissions and reductions of N_2_O on these hilly vineyard lands. Feng et al. [10] found that nodule characteristics can directly enhance nitrogen metabolism, thereby promoting photosynthesis and ultimately having an indirect positive effect on soybean yield. Intercropping soybeans with mulberry can improve soybean yield by optimizing utilization of natural resources. Li et al. [11] examined the structure and variety of microbial communities within alfalfa intercropping systems, exploring the variations in bacterial and fungal populations and their connections to environmental conditions. Their study revealed that intercropping notably reduced soil pH. (The pH values for mulberry and alfalfa monocultures were 7.91 and 7.86, respectively, while the pH value for mulberry-alfalfa intercropping decreased to 7.74). and significantly boosted the total phosphorus levels, although it had no significant impact on the total soil carbon or total nitrogen content. Additionally, intercropping enhanced the relative abundance of *Actinobacteria* in the soil while reducing the relative abundance of Proteobacteria. In intercropped maize systems, alfalfa exhibits greater invasiveness and competitiveness [12]. Intercropping alfalfa enhances the nutrient cycling rate and improves soil and water conservation in agroforestry systems. Ma et al. [13] found that promoting the intercropping of ryegrass and Lingwu jujube in the arid and ecologically fragile northern regions of China can optimize resource utilization and enhance ecosystem resilience. Interactions between trees and alfalfa can establish compensatory and facilitative mechanisms that alter the fundamental physicochemical properties of the soil, increase crop yields, and regulate environmental factors within agroforestry systems to promote the growth of trees. Thus, it can be concluded that intercropping alfalfa under trees is a mutually beneficial cropping pattern with broad application prospects.

Globally, water and fertilizer are key regulatory factors for plant growth and crucial management elements in grassland and agricultural ecosystems [14]. Rational irrigation regimes and fertilization programs can fully leverage the synergistic effects of water and fertilizer, promoting the growth and development of alfalfa as well as its absorption of soil nutrients, thereby increasing alfalfa yield and quality, reducing resource waste, and preventing the deterioration of agricultural environments [15]. Under different water and nitrogen management regimes, there are significant differences in the dry matter and nitrogen accumulation of alfalfa, as well as its ability to absorb and utilize nitrogen. The amount of irrigation water and the application rate of nitrogen fertilizer can influence the physiological functions of crops, leading to differences in the absorption, utilization, and allocation of nitrogen among various plant organs. This ultimately affects the nitrogen-use efficiency and yield accumulation of plants [16]. Ma et al. [17] discovered that optimizing irrigation levels and nitrogen fertilizer application can substantially enhance the yield and crude protein content of alfalfa. Nevertheless, excessive irrigation and nitrogen application beyond optimal levels fail to further boost yields or crude protein content and may cause considerable resource wastage and environmental contamination. Yin et al. [18] optimized the planting method of alfalfa through a nitrogen fertilizer regime based on ridge-film mulching. Their study indicated that ridge-film mulching combined with 160 kg·hm^−1^ of nitrogen fertilizer improved the performance of alfalfa, achieving a balanced enhancement in yield, forage quality, and water and N-use efficiency. However, these studies on water and nitrogen management have primarily focused on the sole cropping of alfalfa, with little mention of intercropping.

Due to the limitations of natural environmental conditions and differences in the level of irrigation agricultural technology [19], current research on agroforestry systems mostly focuses on the ecological functions and benefits of “trees,” while relatively few studies have explored the production effects of intercropped forage under different water and nitrogen management regimes [20]. The Jingtaichuan Power Irrigation District (Jingdian Irrigation District) in Gansu Province is located in the arid and semi-arid sandy belt of northern China. It is a typical high-lift water diversion irrigation district that falls within the ecological suitability zone for alfalfa. Given this [21], this study used alfalfa in the goji berry-alfalfa intercropping system as the research subject, with the hypothesis that a combination of moderate water deficit and optimal nitrogen application can optimize biomass allocation, nitrogen translocation, and nitrogen use efficiency in alfalfa without significantly reducing yield. Through a two-year field experiment, this study explored the response of intercropped alfalfa to water and nitrogen management. Main objectives: (1) To compare the effects of different water and nitrogen management treatments on the allocation of aboveground and belowground biomass in alfalfa; (2) To investigate the changes in nitrogen accumulation and translocation in alfalfa stems and leaves under different irrigation and nitrogen application conditions; (3) To explore the impact of water and nitrogen management on alfalfa hay yield and nitrogen use efficiency; (4) To identify the optimal water and nitrogen management strategy for intercropped alfalfa in the Jingdian Irrigation District, providing a scientific basis for high-yield, water-saving, and sustainable cultivation techniques for alfalfa in this region.

## 2. Results

### 2.1. Effects of Water and Nitrogen Management on Biomass Allocation in Intercropped Alfalfa

#### 2.1.1. Stem-to-Leaf Allocation

In 2022, the results of the analysis of variance showed that the nitrogen application rate and irrigation level had a highly significant effect on the dry weight of alfalfa stems and leaves (*p* < 0.01). The interaction between water and nitrogen significantly affected the dry weights of the first and third crop stems and third crop leaves (*p* < 0.05) (Table 1). Under the same irrigation level, alfalfa stem and leaf dry weights first increased and then decreased with increasing nitrogen application rate. Under the same nitrogen application rate, water deficiency inhibited alfalfa stem growth, with stem dry weights under W1, W2, and W3 being 11.8%, 24.1%, and 25.3% lower on average than under W0, respectively. There was no significant difference in alfalfa leaf dry weight between W0 and W1, while the leaf dry weights under W2 and W3 were, on average, 22.3% and 21.9% lower than those under W0, respectively. From a crop rotation perspective, stem dry weight in the third crop was significantly lower than that in the first two crops, and leaf dry weight decreased gradually with crop rotation.

The stem-to-leaf ratio reflects the distribution of dry matter accumulation between stems and leaves. Irrigation and nitrogen application as single factors had a highly significant effect on the alfalfa stem-to-leaf ratio (*p* < 0.01); however, their combined effect was not significant (Table 2). In 2021, the alfalfa stem-to-leaf ratio (0.704–1.243) showed that the first crop was lower than the second crop, while in 2022, the alfalfa stem-to-leaf ratio (0.649–1.014) showed that the first crop was lower than the third crop, which was lower than the second crop. Under the same irrigation level, the alfalfa stem-to-leaf ratio first decreased and then increased with increasing nitrogen application. The stem-to-leaf ratio was the smallest in N2, with average reductions of 12.4%, 6%, and 7% compared to N0, N1, and N3, respectively. Under the same nitrogen application level, the stem-to-leaf ratio first decreased and then increased with the severity of water deficit. The stem-to-leaf ratio reached its minimum in W1, showing average reductions of 8% relative to W0, 4.5% relative to W2, and 12.9% relative to W3. Among all treatments, W1N2 had the lowest stem-to-leaf ratio, indicating that moderate water deficit combined with moderate nitrogen application is more beneficial for improving alfalfa stem-to-leaf ratio.

#### 2.1.2. Root-Shoot Ratio

**Figure 1:**
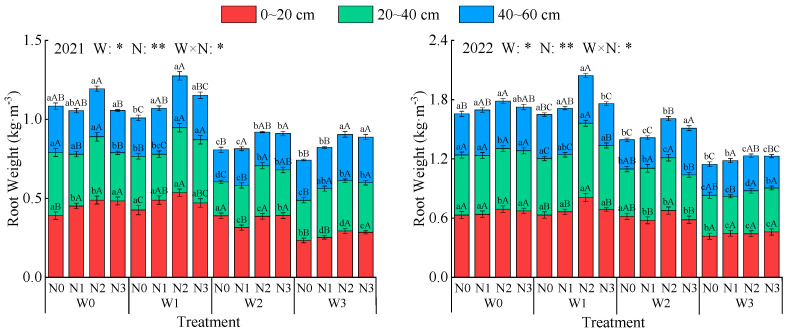
Effects of water and nitrogen regulation on alfalfa root weights. W indicates water regulation, N indicates nitrogen fertilizer regulation, W × N indicates the interaction between the two, * indicates a significant difference (*p* < 0.05), and ** indicates a highly significant difference (*p* < 0.01). Lowercase letters indicate differences among the four irrigation levels at the same depth and the same nitrogen application rate, while uppercase letters indicate differences among the four nitrogen application rates at the same depth and the same irrigation level.

Irrigation, nitrogen application, and their interaction all significantly affected alfalfa root weight (*p* < 0.05, Figure 1). Compared to 2021, the average alfalfa root weight increased by 57.5% in 2022. Under the same irrigation level, root weight showed a trend of first increasing and then decreasing with increasing nitrogen application, reaching a maximum at the N2 level, with average increases of 19.2%, 10.6%, and 7.4% compared to N0, N1, and N3, respectively. Under the same nitrogen application level, root weight first increased and then decreased with the intensification of water deficit, reaching a maximum at the W1 condition, with average increases of 0.9%, 18.6%, and 38.4% compared to W0, W2, and W3, respectively. Root weight distribution showed a decreasing trend with increasing soil depth. The root weights in the 0–20 cm soil layer were 0.393 kg·m^−^^3^ (2021) and 0.603 kg·m^−^^3^ (2022), respectively, accounting for an average of 39.5% of the total, which was 32.62% higher than the average root weight in the 40–60 cm soil layer. Among all treatments, W1N2 had the highest total root weight (1.28 kg·m^−^^3^ in 2021 and 1.91 kg·m^−^^3^ in 2022) and root weights in all soil layers.

**Figure 2:**
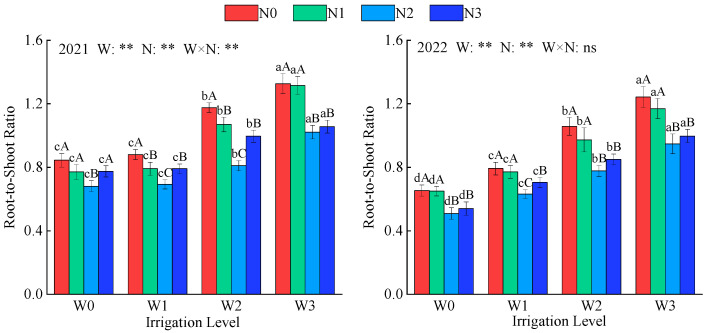
Effects of water and nitrogen regulation on alfalfa root-to-shoot ratios. W indicates water regulation, N indicates nitrogen fertilizer regulation, W × N indicates the interaction between the two, ns indicates no significant difference (*p* > 0.05), and ** indicates a highly significant difference (*p* < 0.01). Lowercase letters indicate differences between the four irrigation levels at the same nitrogen application rate, while uppercase letters indicate differences between the four nitrogen application rates at the same irrigation level.

The root-to-shoot ratio reflects the distribution of dry matter accumulation between the aboveground and belowground parts. Irrigation, nitrogen application, and their combined effects (except in 2022) significantly impacted the root-to-shoot ratio of alfalfa (Figure 2, *p* < 0.01). In 2022, the root-to-shoot ratio decreased by 11.5% compared to that in 2021. Under the same irrigation level, the root-to-shoot ratio first decreased and then increased with increasing nitrogen application levels, reaching a minimum at N2, which was on average 23.9%, 19.2%, and 9.5% lower than N0, N1, and N3, respectively. Under the same nitrogen application level, the root-to-shoot ratio increased as the irrigation deficit worsened, reaching its minimum at W0, with average decreases of 10.4%, 29.6%, and 40.5% compared to W1, W2, and W3, respectively. The root-to-shoot ratio of alfalfa was lowest under the W0N2 treatment (0.681 and 0.509) and highest under the W3N0 treatment (1.33 and 1.24).

### 2.2. The Effect of Water and Nitrogen Regulation on Nitrogen Turnover in Intercropped Alfalfa

#### 2.2.1. Alfalfa Nitrogen Accumulation

**Figure 3:**
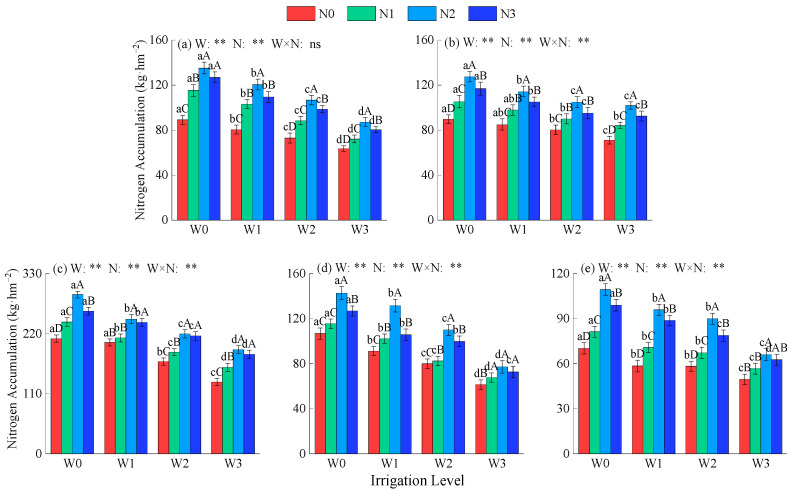
Effects of water and nitrogen regulation on nitrogen accumulation in alfalfa. (**a**,**b**) represent the first and second crops of alfalfa in 2021, respectively, while (**c**–**e**) represent the first, second, and third crops of alfalfa in 2022, respectively. W indicates water regulation, N indicates nitrogen fertilizer regulation, W × N indicates the interaction between the two, ns indicates no significant difference (*p* > 0.05), and ** indicates a highly significant difference (*p* < 0.01). Lowercase letters indicate differences between the four irrigation levels at the same nitrogen application rate, while uppercase letters indicate differences between the four nitrogen application rates at the same irrigation level.

Irrigation, nitrogen application, and their interactive effects (excluding the first crop in 2021) had a highly significant impact on alfalfa nitrogen accumulation (Figure 3; *p* < 0.01). Overall, the total nitrogen accumulation in alfalfa under all treatments in 2022 was significantly higher than that in 2021, and as the alfalfa crop progressed, nitrogen accumulation showed a gradually decreasing trend. Under the same irrigation level, nitrogen accumulation first increased and then decreased with increasing nitrogen application rates, reaching a peak under N2 conditions, with average increases of 39.6%, 21.4%, and 8.6% compared to N0, N1, and N3, respectively. Under the same nitrogen application rate, nitrogen accumulation decreases as irrigation deficit severity increases. Compared with W0, W1, W2, and W3, nitrogen accumulation increased by an average of 12.1%, 25.9%, and 51%, respectively. Nitrogen accumulation reached its peak under W0N2, doubling that of the W3N0 treatment.

**Figure 4:**
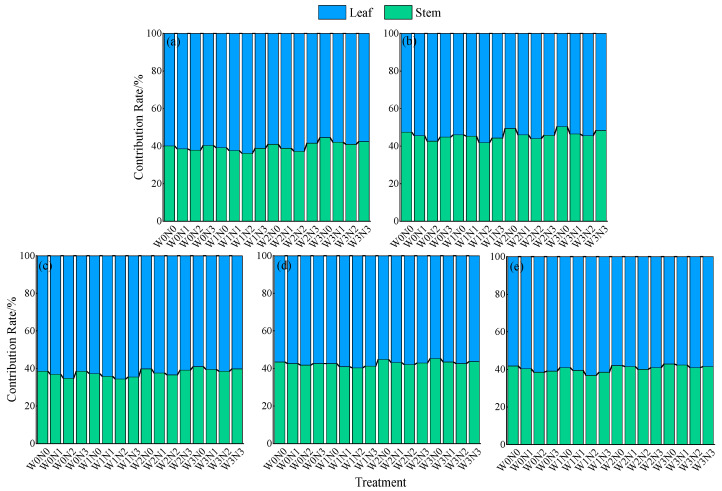
Effects of water and nitrogen regulation on the nitrogen contribution rate of alfalfa stem and leaf. (**a**,**b**) represent the first and second crops of alfalfa in 2021, respectively, while (**c**–**e**) represent the first, second, and third crops of alfalfa in 2022, respectively.

Under different water-nitrogen regulation conditions, the nitrogen contribution rate of leaves was, on average, 17.4% higher than that of stems (Figure 4). Over the two years, the nitrogen contribution rate of leaves in 2021 showed a pattern of first crop > second crop, while in 2022, it showed a pattern of first crop > third crop > second crop. The nitrogen contribution rate of stems exhibited the opposite pattern. Under the same irrigation level, the nitrogen contribution rate of leaves first increased and then decreased with increasing nitrogen application rates. Under the same nitrogen application rate, the nitrogen contribution rate of leaves first increased and then decreased as water deficit intensified. The nitrogen contribution rate of leaves in W1 was slightly higher, with no significant difference from that in W0. Among all treatments, W1N2 had the highest leaf contribution rate, averaging 62.1%.

#### 2.2.2. Stem and Leaf Nitrogen Transport

Irrigation, nitrogen application, and their interactive effects all significantly influenced alfalfa nitrogen transport (*p* < 0.05, Table 3). Overall, nitrogen transport in 2022 increased by an average of 61.5% compared to 2021 and showed a decreasing trend as crop cycles progressed. Under the same irrigation level, nitrogen transport initially increased and then decreased as the nitrogen application rate increased. Under N2, nitrogen transport was the highest, with average increases of 23% over N0, 5.4% over N1, and 10.3% over N3. Under the same nitrogen application level, nitrogen transport first increased and then decreased as water deficit intensified, with the highest values under W1 conditions, increasing by an average of 4.7%, 17.3%, and 25.3% compared to W0, W2, and W3, respectively. Among all treatments, W1N2 achieved the highest nitrogen transport rate, with values of 75.6 kg·hm^−^^2^ and 195 kg·hm^−^^2^ in the two years, respectively, representing average increases of 15.1% and 33.4% compared to W0N0 and W3N0.

#### 2.2.3. Soil Nitrogen Balance

Based on existing research and experimental data [22,23,24], the estimated nitrogen balance results are presented in Table 4. There were differences in soil nitrogen changes under different treatments. The soil nitrogen changes under W0N0 and W1N0 were −20 kg·hm^−^^2^ and −10 kg·hm^−^^2^, respectively, indicating that nitrogen output was slightly greater than the input. Under the same irrigation level, the change in soil nitrogen increases with increasing nitrogen application rate. Under the same nitrogen application rate, the change in soil nitrogen gradually increases with the severity of water deficit. Under W3N3 treatment, the maximum change in soil nitrogen was 167.5 kg·hm^−^^2^.

### 2.3. The Effect of Water and Nitrogen Regulation on the Production of Intercropped Alfalfa

#### 2.3.1. Hay Yield

**Figure 5:**
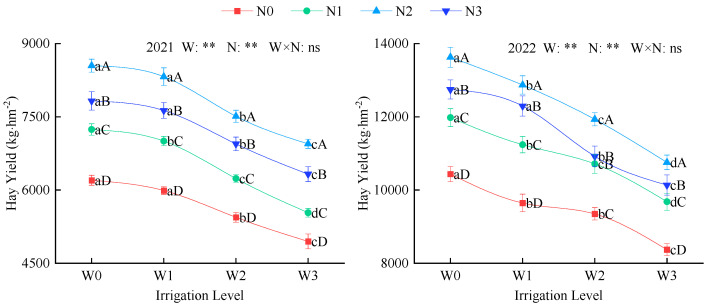
Effects of water and nitrogen regulation on alfalfa hay yield. W indicates water regulation, N indicates nitrogen fertilizer regulation, W × N indicates the interaction between the two, ns indicates no significant difference (*p* > 0.05), and ** indicates a highly significant difference (*p* < 0.01). Lowercase letters indicate differences between the four irrigation levels at the same nitrogen application rate, while uppercase letters indicate differences between the four nitrogen application rates at the same irrigation level.

Irrigation and nitrogen application had a highly significant effect on alfalfa hay yield (*p* < 0.01; Figure 5). Hay yield increased by an average of 62.63% in 2022 (8373–12,872 kg·hm^−^^2^) compared to 2021 (4949–8325 kg·hm^−^^2^). Under the same irrigation level, hay yield increased with increasing nitrogen application, following the order N2 > N3 > N1 > N0. Compared with N0, N1, and N2, N2 showed an average increase of 33.3%, 15.6%, and 7.6%, respectively. At the same nitrogen application rate, hay yield increased with increasing irrigation volume, following the order of W0 > W1 > W2 > W3. W0 was, on average, 4.8%, 13.8%, and 25.4% higher than W1, W2, and W3, respectively. Among all treatments, W0N2 had the highest yield, with average increases of 42% and 67.8% compared to W1N0 and W3N0, respectively.

#### 2.3.2. Nitrogen Use Efficiency

**Figure 6:**
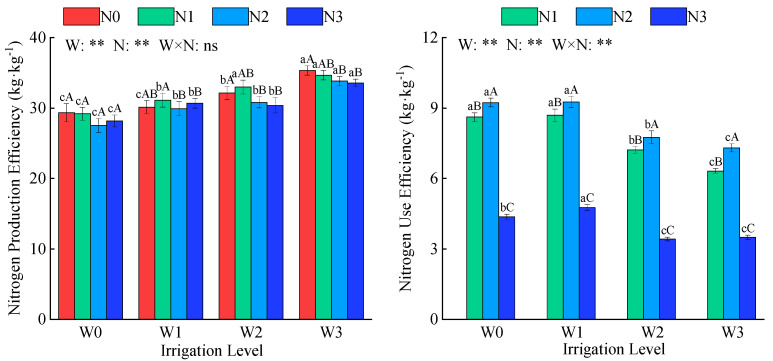
Effects of water and nitrogen regulation on alfalfa nitrogen production efficiency and nitrogen use efficiency. W indicates water regulation, N indicates nitrogen fertilizer regulation, W × N indicates the interaction between the two, ns indicates no significant difference (*p* > 0.05), and ** indicates a highly significant difference (*p* < 0.01). Lowercase letters indicate differences between the four irrigation levels at the same nitrogen application rate, while uppercase letters indicate differences between nitrogen application rates at the same irrigation level.

Irrigation and nitrogen application had a significant effect on alfalfa nitrogen production and utilization efficiency (*p* < 0.01, Figure 6). Under the same irrigation conditions, nitrogen production efficiency was highest at the N1 application level, with an average value of 32 kg·kg^−^^1^. Under the same nitrogen application rate, nitrogen production efficiency increased gradually as water deficit severity increased, with W3 showing an average increase of 20.4% compared with W0. Alfalfa nitrogen production efficiency was highest in the W3N0 treatment. Under the same irrigation level, nitrogen utilization efficiency first increased and then decreased with increasing nitrogen application rate, showing the following order: N2 > N1 > N3, with N2’s nitrogen utilization efficiency averaging twice that of N3. At the same nitrogen application rate, nitrogen utilization efficiency first increased and then decreased as water deficit severity increased, in the following order: W1 > W0 > W2 > W3. The nitrogen utilization efficiency of W1 was 32.7% higher than that of W3. Among all treatments, alfalfa nitrogen utilization efficiency reached its maximum value of 9.3 kg·kg^−^^1^ under the W1N2 treatment.

### 2.4. Comprehensive Evaluation of the Effects of Water and Nitrogen Regulation on Intercropped Alfalfa

**Figure 7:**
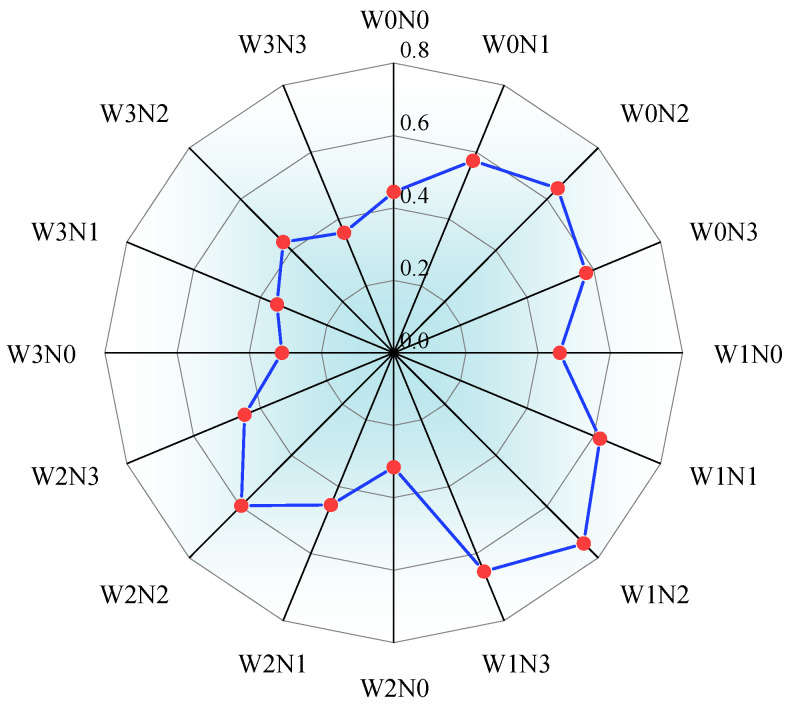
Comprehensive scores for each alfalfa treatment calculated using the TOPSIS method.

A comprehensive evaluation analysis of various indicators of alfalfa under different water and nitrogen regulation conditions was conducted using the entropy weight-TOPSIS method. As shown in Table 5, root weight (17.2%) and nitrogen production efficiency (14.7%) had the highest information dispersion and thus the highest weights, exerting the most significant influence on the evaluation results. The next most important factors were nitrogen accumulation (12.6%) and root-to-shoot ratio (12.2%). Hay yield (12%) and nitrogen transport rate (11.3%) had similar weights, and their information entropy values maintained a moderate influence. The leaf nitrogen contribution rate (10.4%) and stem-to-leaf ratio (9.6%) have the lowest weights, consistent with their high information entropy values (0.947 and 0.951), indicating that the data distribution tends toward homogenization. Using the TOPSIS method to analyze the weighted data (Figure 7), the W1N2 treatment had the highest comprehensive score (0.744), indicating the best overall benefit. W1N3 and W0N2 followed, while W3N0 had the lowest comprehensive score (0.309).

## 3. Discussion

### 3.1. The Effect of Water and Nitrogen Regulation on Alfalfa Growth and Yield

Hay yield reflects the total biomass of aboveground organs produced by forage crops through photosynthesis per unit area. Water and nitrogen are important factors that influence crop biomass formation [25]. Compared to other crops, alfalfa is considered a high-water-demand crop [26], and its yield is highly sensitive to soil moisture supply. Adequate irrigation is crucial for enhancing alfalfa productivity. Wang et al. [27] found that drought stress causes alfalfa to grow slowly, resulting in shorter plants and reduced yields. As irrigation volume increases, plant cell turgor pressure rises, photosynthesis intensifies, and the yield of assimilates increases. This study found that alfalfa yield increases with increasing irrigation volume, with significant differences in yield between W0 and W1 conditions and W3. This is because soil moisture under adequate irrigation conditions falls within the optimal range for alfalfa growth, consistent with the findings of Liu [28], Li [29], and others. The difference in hay yield between W0 and W1 was not significant, indicating that irrigation at 65–75% of local field capacity can maintain normal alfalfa yield. Therefore, from an irrigation perspective, local management of alfalfa cultivation grasslands can adopt mild water deficit irrigation, which can appropriately conserve farm irrigation water while avoiding significant yield reduction.

When irrigation levels are optimal, water is no longer a limiting factor for yield, and yield increases are constrained by other factors, such as soil fertility [30]. In the Ningxia region, [31] different irrigation and fertilization treatments were applied, and the results showed that both had a significant impact on alfalfa yield formation. Liu et al. [32] studied the water and fertilizer effects on alfalfa and found that irrigation treatments had a greater impact on alfalfa yield than fertilization treatments. Appropriate irrigation volumes are beneficial for increasing dry hay yields in the first year of alfalfa cultivation. Ren et al. [33] analyzed data using the Aggregated Boosted Tree (ABT) algorithm, showing that nitrogen fertilizer application contributed up to 22.55% to annual yield. The total yield of alfalfa dry hay initially increased and then decreased with increasing fertilizer application, following the law of diminishing returns. This study also reached similar conclusions, with total dry matter yields of alfalfa under N1 and N2 conditions being higher than those under N0 and N3 conditions. This may be due to a threshold in nitrogen application rates, where low and moderate nitrogen application rates can meet the growth requirements of alfalfa during specific growth stages, facilitating the accumulation of photosynthetic products, while no nitrogen application or high nitrogen application rates limit the formation of alfalfa biomass. Water serves as a carrier for fertilizer dissolution and migration, and fertilizers can only move and be transported within the soil when dissolved in water, thereby being absorbed and utilized by crops [34]. Under drought stress, water becomes a limiting factor affecting alfalfa yield, while under adequate water conditions, alfalfa yield is constrained by fertilizer availability. Water and fertilizer do not act independently but interact synergistically to enhance each other’s effects [35]. It is evident that an appropriate water and nitrogen supply is an effective approach to enhance alfalfa yield.

In addition, hay yield is influenced by factors such as climate, random sampling, and the drying process [36]. Alfalfa has a strong regrowth capacity and can be harvested multiple times during its growing season. However, as harvest time progresses, hay yield from each cutting shows a significant decline. This study found that the first cutting yielded the highest hay production, while the last cutting yielded the lowest, consistent with most previous studies [37]. The primary reason for this phenomenon is closely related to the ability of alfalfa to recover after cutting. Changes in meteorological conditions, such as temperature and daylight hours, can significantly impact alfalfa growth in the later stages [38]. Additionally, differences in planting regions, climate, and alfalfa varieties result in varying hay yields, even among the same alfalfa variety. Therefore, planting management must be adjusted according to the actual conditions of the alfalfa cultivation region [39].

### 3.2. The Effect of Water and Nitrogen Regulation on Nitrogen Allocation in Alfalfa Plants

The stems and leaves of alfalfa are the primary edible parts, and the distribution of nutrients between the stems and leaves is crucial for improving alfalfa quality [40]. The stem-to-leaf ratio reflects the forage utilization value and the accumulation of dry matter [41], making it an important indicator for assessing alfalfa production performance, planting efficiency, and economic value. A smaller alfalfa stem-to-leaf ratio indicates a higher leaf yield, better palatability for livestock during feeding, and higher alfalfa quality. Different irrigation levels significantly affected the alfalfa stem-to-leaf ratio, with the smallest ratio observed under W1 conditions, which was significantly different from that under other treatments. No significant difference was observed between W0 and W1, but a significant difference was observed between W0 and W3. Different nitrogen application levels also significantly affect the alfalfa stem-to-leaf ratio, with the smallest ratio under N2 conditions, showing significant differences compared to N0, N1, and N3. However, under conditions of low irrigation (W3) or no nitrogen application (N0), the alfalfa stem-to-leaf ratio becomes excessively high, resulting in a correspondingly lower quality. Under different water-nitrogen combinations, the stem-to-leaf ratio of alfalfa was lowest under W1N2 conditions. Zhang et al. [42] found that water addition reduces the stem-to-leaf ratio of alfalfa, thereby improving its quality; Ma et al. [43] found that although increased water promotes alfalfa growth, the leaf proportion does not increase, and the nitrogen content of alfalfa plants does not improve. In 2021, the alfalfa stem-to-leaf ratio of the first crop was lower than that of the second crop, which was determined by the natural growth patterns of alfalfa. After alfalfa enters the late growth stage, photosynthetic products in the leaves are continuously transferred to the flower buds and stems [44], and the accumulation rate of leaf dry matter is lower than that of the stems. The relative weight of alfalfa leaves gradually decreases, and the stem-to-leaf ratio shows an upward trend [45]. In 2022, the stem-to-leaf ratio of alfalfa was lower in the first crop than in the third crop and higher than that in the second crop. This could be attributed to the substantial decline in meteorological factors like temperature and daylight hours, resulting in a significant reduction in dry matter accumulation in the third crop of alfalfa, with both leaf dry weight and stem dry weight decreasing significantly, and tender branches leading to a low stem-to-leaf ratio. Leaves are the primary nutritional organs of alfalfa [46], and under different water-nitrogen management conditions, the nitrogen contribution rate of alfalfa leaves remained around 60%. This may be because leaves typically have a larger specific surface area and higher metabolic activity, enabling them to absorb, fix, and utilize nitrogen more efficiently. In contrast, stems have a denser structure with a smaller specific surface area, resulting in slower nitrogen absorption and metabolic rates, and thus a lower nitrogen contribution rate.

This study found that alfalfa root weight was highest under W1N2 conditions, indicating that mild water deficit and moderate nitrogen application can mitigate the effects of water deficit on alfalfa growth by promoting root development [47]. However, root weight under N3 conditions was lower than that under N2 conditions, suggesting that excessive nitrogen application is detrimental to alfalfa growth and development. The root-to-shoot ratio, representing the biomass proportion between the underground and aboveground portions of alfalfa, can indicate the plant’s growth strategies and physiological adaptations under varying conditions. With increasing nitrogen application, the root-to-shoot ratio initially declines before rising. The root-to-shoot ratio did not significantly differ between W0 and W1 but was notably lower in W3. This suggests that severe water deficiency negatively impacts alfalfa growth. Irrigation, nitrogen application, and their interactive effects significantly influence nitrogen accumulation in alfalfa, with the highest nitrogen accumulation observed under W0N2 conditions. The nitrogen transport rate at harvest also varied with different irrigation and nitrogen application levels, being higher under W0 and W1, indicating that water supply is the foundation for promoting nitrogen absorption and transport, and an adequate water supply can fully utilize efficient nitrogen absorption and optimize its distribution [48]. The nitrogen transport rate showed a trend of first increasing and then decreasing with increasing nitrogen application, reaching its maximum under N2 conditions. The results indicate that both alfalfa nitrogen accumulation and transport require an appropriate water-nitrogen combination to ensure optimal outcomes.

### 3.3. The Effect of Water and Nitrogen Regulation on Nitrogen Absorption and Utilization in Alfalfa

In arid regions where water and nitrogen resources are increasingly scarce, achieving high crop yields must be accompanied by efficient utilization of water and fertilizer resources [49]. An optimal water-nitrogen management model is crucial not only for determining whether crops can achieve high yields but also for enhancing the efficiency of water and nitrogen use [50]. As an excellent leguminous forage crop, determining the appropriate water and nitrogen application rates is a key factor for increasing alfalfa yields [51]. This study found that alfalfa nitrogen utilization efficiency first increases and then decreases with increasing nitrogen application rates. This indicates that under low nitrogen application conditions, alfalfa has a high demand for nitrogen fertilizer, which not only leads to more intense competition for nitrogen absorption [52] but also promotes increased atmospheric nitrogen fixation and improves nitrogen use efficiency. When the nitrogen demand of alfalfa reaches saturation, excessive nitrogen fertilizer exceeds the plant’s nitrogen demand and utilization capacity, leading to excessive nitrogen absorption and reduced nitrogen fertilizer utilization efficiency. Liu et al. [53] analyzed the planting costs and economic benefits of alfalfa, wheat, and corn. Considering planting costs, alfalfa has the highest yield, and promoting its cultivation can bring significant economic benefits to the local area. Optimizing the match between nitrogen fertilizer supply and crop nitrogen demand can reduce nitrogen losses. As irrigation levels decrease, nitrogen absorption by plant organs also decreases; however, mild water deficit irrigation does not affect alfalfa nitrogen absorption or utilization. Therefore, it is recommended to establish a treatment combining moderate nitrogen application and mild water deficit irrigation (W1N2) during alfalfa production to achieve efficient nitrogen utilization.

## 4. Materials and Methods

### 4.1. Description of the Experimental Site

**Figure 8:**
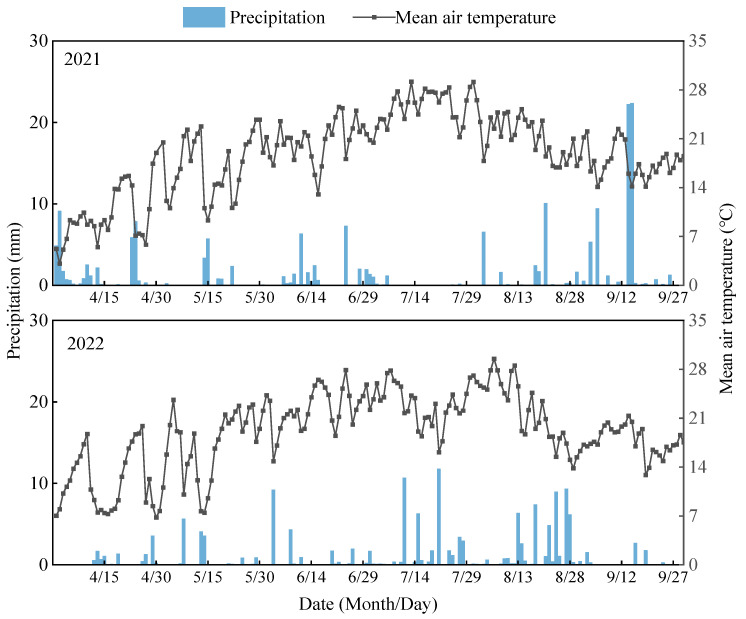
Distribution of precipitation and temperature in the alfalfa growth period test area.

The experiment was conducted from April to October in 2021 and 2022 at the Jingtaichuan Power Irrigation Water Resources Utilization Center Irrigation Experiment Station in Gansu Province (37°23′ N, 104°08′ E, elevation 1563 m). The site experiences a temperate continental arid climate with 2652 h of annual sunshine, a 191-day frost-free period, an average annual temperature of 8.5 °C, and average annual precipitation and evaporation of 185 mm and 3028 mm, respectively. The soil type is sandy loam, and its basic physicochemical properties within the 0–100 cm layer are listed in Table 6. The distributions of precipitation and temperature during the alfalfa growing season are shown in Figure 8.

### 4.2. Experimental Design

**Figure 9:**
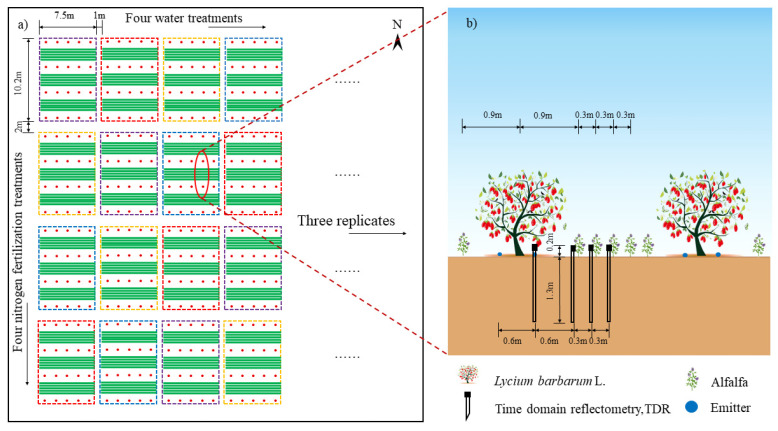
Layout of the test plots. (**a**) shows the layout of the experimental plot, and (**b**) shows the planting pattern.

The experiment was conducted on 4 April 2021, by transplanting two-year-old “Ningqi No. 5” goji berry saplings with thick main stems, a similar number of branches, and no pests or diseases. The saplings were planted in east-west rows with a spacing of 1.5 m × 3 m. Alfalfa was broadcast-sown in bare soil between the goji berry rows, 90 cm east-west from the goji berry tree trunks. The alfalfa variety used was “Longdong Alfalfa,” with a seeding rate of 13 kg·hm^−2^ and a row spacing of 30 cm (Figure 9). After establishment, both goji berries and alfalfa were thoroughly irrigated, and water and nitrogen management began in June. Alfalfa was harvested twice in 2021 and three times in 2022. The growth periods of each alfalfa crop are shown in Table 7.

This study utilized a randomized complete block design with two factors. Soil moisture was managed as a percentage of field capacity (θ_fc_) to determine the upper and lower bounds of soil water content based on local farming practices. Four irrigation regimes were defined: full irrigation (W0, 75–85% θ_fc_), mild water deficit (W1, 65–75% θ_fc_), moderate water deficit (W2, 55–65% θ_fc_), and severe water deficit (W3, 45–55% θ_fc_). Within each irrigation regime, four nitrogen application rates were tested: no nitrogen (N0, 0 kg·hm^−2^), low nitrogen (N1, 150 kg·hm^−2^), medium nitrogen (N2, 300 kg·hm^−2^), and high nitrogen (N3, 450 kg·hm^−2^). This resulted in 16 treatment combinations (Table 8). Each treatment has three replicates, with a plot area of 76.5 m^2^ (10.2 m × 7.5 m).

Drip irrigation was used, with drip tape spaced at 0.3 m intervals, a designed emitter flow rate of 2.0 L·h^−1^, and emitter spacing of 0.3 m. Each plot was equipped with valves and water meters (accuracy 0.0001 m^3^) to control the amount of water applied. Prior to irrigation, soil moisture levels were assessed using a portable time-domain reflectometer (TDR; PICO-BT, IMKO, Germany), and the irrigation volume was calculated based on the difference between the current and target soil moisture contents for each treatment, as well as the planned wetting depth of 60 cm, to ensure that the soil moisture content after irrigation remained within the designed moisture range for each treatment. The fertilizers employed in the experiment included urea (46% N content), superphosphate (12% P_2_O_5_), and potassium sulfate (60% K_2_O content). The applied P and K doses were 57.2 kg·hm^−2^ and 108 kg·hm^−2^, respectively, along with 60% of the nitrogen fertilizer, which was applied at the time of establishment. The remaining 40% of the nitrogen fertilizer was applied as a top dressing before the regrowth of alfalfa in the following season using a Venturi fertilizer injector. The edges of the experimental plots were lined with 2 m deep plastic membranes to prevent the lateral movement of water and nutrients. Walkways 2 m and 1 m wide were set between experimental groups and plots, respectively. Before the treatments began, the plots were uniformly irrigated to their field capacity. Subsequently, the experimental treatments were initiated. Weeding, pruning, and pest and disease control were performed in accordance with local practices.

### 4.3. Sample Collection and Analysis Methods

#### 4.3.1. Biomass

Throughout the branching, budding, and early flowering stages of each alfalfa crop, a 25 cm sample segment was collected from the middle strip of each plot. Fresh forage samples from each treatment were brought to the laboratory for manual separation of stems and leaves (including inflorescences within the leaves). Fresh forage samples were placed in an oven at 105 °C for 30 min to kill the green tissue and then dried at 75 °C until a constant weight was achieved. The dry weights of the stems and leaves were measured, and the stem-to-leaf ratio was calculated.

Following the final alfalfa harvest each year, a small quadrat measuring 25 cm × 15 cm × 60 cm was randomly selected from each plot. Root samples from the 0–60 cm soil layer were collected at 20 cm intervals. The samples were sieved through a 0.5 mm mesh to remove adhering soil particles. The roots were subsequently washed to eliminate residual soil, dried in an oven at 75 °C until they reached a constant weight, and then weighed after cooling.

The formulas for calculating the stem-to-leaf ratio and root-to-shoot ratio are as follows:

Stem-to-leaf ratio (g·g^−1^) = Stem dry weight (g)/Leaf dry weight (g) (1)

Root-to-shoot ratio (g·g^−1^) = Belowground biomass (g)/Aboveground total biomass (g) (2)

#### 4.3.2. Yield

Harvesting and yield measurements were conducted at the early flowering stage of alfalfa (when 1/10 of the plants were in bloom). A quadrat of 1.5 m^2^ (1 m × 0.3 m × 5 rows) was selected in each plot, with a stubble height of 5 cm. Fresh weight was measured immediately after field sampling. Fresh forage was initially placed in an oven at 105 °C for 5 min to deactivate enzymes, then dried at 75 °C until reaching a constant weight. The dry weight of the hay was then determined.

#### 4.3.3. Nitrogen-Related Indicators

After drying and grinding the plant samples(blanching in an oven at 105 °C for 5 min, then drying at 75 °C until a constant weight was achieved) from each growth stage through a 0.25 mm sieve, the total nitrogen content in the stems and leaves was determined using the Kjeldahl method after digestion with H_2_O_2_ and concentrated H_2_SO_4_. The formulas for calculating nitrogen accumulation, contribution rate, translocation, and nitrogen fertilizer production efficiency are as follows:

Nitrogen accumulation (kg·hm^−2^) = Biomass × Nitrogen concentration (3)

Contribution rate = (Nitrogen accumulation in leaves (stems)/Total nitrogen accumulation in leaves and stems) × 100%(4)

Nitrogen translocation (kg·hm^−2^) = Nitrogen accumulation at initial flowering stage-Nitrogen accumulation at budding stage (5)

Nitrogen production efficiency (kg·kg^−1^) = Hay yield (kg·hm^−2^)/Nitrogen uptake by aboveground biomass (kg·hm^−2^) (6)

Nitrogen use efficiency (kg·kg^−1^) = [Hay yield with nitrogen application (kg·hm^−2^)-Hay yield without nitrogen application (kg·hm^−2^)]/Nitrogen application rate (kg·hm^−2^) (7)

Under deficit irrigation, nitrogen leaching is negligible. The nitrogen balance formula is estimated as follows [54]:

Soil nitrogen change = (fertiliser nitrogen + nitrogen fixation)-(nitrogen uptake by alfalfa + nitrogen uptake by goji berries)-gaseous losses(8)

### 4.4. Entropy Weight-TOPSIS Model

#### 4.4.1. Determining the Weights of Indicators Using the Entropy Weight Method

Given m evaluation objects and n evaluation indicators, the values of each evaluation indicator *Y_ij_* (*i* = 1, 2, 3, …, *m*; j = 1, 2, 3, …, *n*) are normalized to obtain the normalized indicator values *X_ij_*, and the weights *W_ij_* of each indicator are calculated as follows:

(9) $$P_{\mathrm{ij}}=\frac{X_{\mathrm{ij}}}{\Sigma_{i=1}^{m}X_{\mathrm{ij}}}$$

(10) ej=−[ln m]−1×Σi=1m[Pij×ln Pij]

(11) $$d_{j}=1-e_{j}$$

(12) $$W_{j}=\frac{1-e_{j}}{\Sigma_{j=1}^{n}\left(1-e_{j}\right)}$$

where *P_ij_* is the contribution degree of the *i*-th evaluation object for the *j*-th indicator, *e_j_* is the information entropy value, *d_j_* is the information utility value, and *W_j_* is the weight obtained for each indicator (%).

#### 4.4.2. Comprehensive Evaluation Using the TOPSIS Method

(1) Construction of the weighted normalized matrix

(13) $$R_{\mathrm{ij}}=W_{j}\times X_{\mathrm{ij}}$$

(2) Determination of the positive ideal solution *R*^+^ and the negative ideal solution *R*^−^

(14) $$R^{+}=\max(R_{i1},R_{i2},\ldots ,R_{\mathrm{im}})$$

(15) $$R^{-}=\min(R_{i1},R_{i2},\ldots ,R_{\mathrm{im}})$$

(3) Optimal solution distance *D*^+^ and worst solution distance *D*^−^

(16) $$D^{+}=\sqrt{\Sigma_{j=1}^{n}{(R_{\mathrm{ij}}-{R_{j}}^{+})}^{2}}$$

(17) $$D^{-}=\sqrt{\Sigma_{j=1}^{n}{(R_{\mathrm{ij}}-{R_{j}}^{-})}^{2}}$$

(4) Relative Closeness *Ci*

(18) $$C_{i}=\frac{D_{i}^{-}}{D_{i}^{+}+D_{i}^{-}}$$

### 4.5. Data Processing and Statistical Analysis

The experimental data were first analyzed using Excel 2021. Two-way ANOVA and multiple comparisons (Duncan’s test) were conducted for each parameter using SPSS 25.0. Data visualization was performed using OriginPro 2022b.

## 5. Conclusions

Irrigation and nitrogen application significantly impact alfalfa production in goji berry-alfalfa intercropping systems. Optimal water and nitrogen management can enhance alfalfa dry matter yield, plant nitrogen accumulation, nitrogen transport, and utilization efficiency while reducing the stem-to-leaf and root-to-crown ratios. Compared to the W0N2 treatment, the W1N2 treatment showed no significant differences in alfalfa root-to-shoot ratio, dry matter yield, and nitrogen utilization efficiency, but nitrogen production efficiency increased by 8.7%, and the alfalfa stem-to-leaf ratio decreased by 8%. The results of the entropy-weighted TOPSIS comprehensive evaluation indicate that maintaining a soil field water-holding capacity of 65–75% and applying 300 kg·hm^−^^2^ of nitrogen (W1N2) is the optimal water-nitrogen management mode for alfalfa intercropping in the Jingdian Irrigation District and similar inland arid regions. This study has significant implications for the development of the alfalfa industry, providing new insights into water and nitrogen management for alfalfa in arid and semi-arid regions, as well as innovative management models for the sustainable development of forest-grass intercropping systems.

## Figures and Tables

**Table 1:** Effect of water and nitrogen regulation on stem and leaf dry weights of intercropped alfalfa.

Treatment	Stem Dry Weight (g)			Leaf Dry Weight (g)		
	1st Harvest	2nd Harvest	1st Harvest	1st Harvest	2nd Harvest	1st Harvest
W0N0	17.2 ± 0.75 aA	18.3 ± 0.75 aC	14.9 ± 0.85 aC	21.2 ± 1.03 aC	18.5 ± 0.54 aC	16.8 ± 1.23 aC
W0N1	17.5 ± 0.85 aA	19.6 ± 0.85aBC	17.2 ± 0.45aB	23.7 ± 0.84aAB	21.1 ± 1.27aB	19.8 ± 0.81aB
W0N2	18.2 ± 0.55 aA	21.7 ± 0.91 aA	19.8 ± 0.79 aA	25.6 ± 1.42 aA	24.3 ± 0.95 aA	24.2 ± 0.72 aA
W0N3	18.1 ± 0.84 aA	20.9 ± 0.84 aAB	19.4 ± 0.42 aA	22.9 ± 0.98 aBC	22.8 ± 1.25 aAB	22.9 ± 1.35 aA
W1N0	15.5 ± 0.67 bAB	16.8 ± 1.40 aB	13.3 ± 0.55 bC	21.2 ± 0.80 aB	18.4 ± 0.77 aC	15.7 ± 0.76 aC
W1N1	16.4 ± 0.65 aA	17.8 ± 0.55 bAB	14.8 ± 0.71 bB	23.7 ± 0.73 aA	20.9 ± 1.32 aB	18.5 ± 1.12 aB
W1N2	16.8 ± 0.65 bA	19.6 ± 1.35 bA	16.5 ± 0.65 bA	25.9 ± 1.84 aA	23.6 ± 1.35 aA	22.9 ± 0.63 aA
W1N3	14.5 ± 0.75 bB	17.8 ± 0.70 bAB	16.8 ± 0.87 bA	21.3 ± 1.21 aB	20.7 ± 0.82 aB	21.9 ± 0.92 aA
W2N0	12.8 ± 0.37 cA	14.8 ± 0.38 bB	11.7 ± 0.75 cB	16.7 ± 1.03 bB	15.7 ± 0.36 bB	13.3 ± 0.67 bB
W2N1	13.4 ± 0.9 bA	16.0 ± 0.56 cAB	13.3 ± 0.95 cA	18.8 ± 1.04 bAB	17.6 ± 1.41 bAB	16.1 ± 0.99 bA
W2N2	13.5 ± 0.6 cA	17.0 ± 1.25 cA	13.5 ± 0.65 cA	20.5 ± 0.96 bA	19.3 ± 0.59 bA	17.6 ± 0.9 bA
W2N3	13.7 ± 0.71 bA	15.8 ± 0.74 cAB	13.6 ± 0.45 cA	17.8 ± 1.66 bB	17.3 ± 1.28 bAB	17.2 ± 0.66 bA
W3N0	11.7 ± 0.82 cB	14.1 ± 0.49 bB	11.3 ± 0.4 cC	13.8 ± 0.74 cC	13.9 ± 0.83 cB	12.2 ± 0.9 bC
W3N1	13.3 ± 0.63 bA	15.1 ± 0.45 cAB	13.3 ± 0.35 cB	16.6 ± 0.79 cB	16.1 ± 1.14 bA	14.8 ± 0.45 bB
W3N2	14.6 ± 0.7 cA	16.0 ± 0.45 cA	14.4 ± 0.83 cA	18.9 ± 0.67 bA	17.6 ± 0.71 bA	16.9 ± 1.26 bA
W3N3	13.9 ± 0.75 bA	15.4 ± 1.13 cAB	13.6 ± 0.45 cAB	17.2 ± 0.95 bB	16.2 ± 1.02 bA	15.7 ± 1.17 bAB
ANOVA						
W	**	**	**	**	**	**
N	**	**	**	**	**	**
W × N	*	ns	**	ns	ns	*

Note: W indicates water regulation, N indicates nitrogen fertilizer regulation, W × N indicates the interaction between the two, ns indicates no significant difference (*p* > 0.05), * indicates a significant difference (*p* < 0.05), and ** indicates a highly significant difference (*p* < 0.01). Lowercase letters indicate differences between the four irrigation levels at the same nitrogen application rate, while uppercase letters indicate differences between the four nitrogen application rates at the same irrigation level.

**Table 2:** Stem-to-leaf ratio of alfalfa under different water and nitrogen regulations.

Treatment	2021		2022		
	1st Harvest	2nd Harvest	1st Harvest	2nd Harvest	3rd Harvest
W0N0	0.853 ± 0.032 bA	1.192 ± 0.028 aA	0.811 ± 0.044 abA	0.989 ± 0.041 abA	0.887 ± 0.045 aA
W0N1	0.779 ± 0.043 bB	1.041 ± 0.046 aB	0.738 ± 0.029 abBC	0.929 ± 0.048 aAB	0.869 ± 0.027 bA
W0N2	0.731 ± 0.031 bB	0.963 ± 0.021 abB	0.711 ± 0.041 abC	0.893 ± 0.046 aB	0.818 ± 0.036 abA
W0N3	0.872 ± 0.035 aA	1.029 ± 0.062 bB	0.79 ± 0.032 aAB	0.917 ± 0.039 abAB	0.847 ± 0.031 abA
W1N0	0.795 ± 0.044 bA	1.035 ± 0.026 bA	0.731 ± 0.035 cA	0.913 ± 0.034 bA	0.847 ± 0.041 aA
W1N1	0.743 ± 0.04 bAB	1.011 ± 0.023 aA	0.692 ± 0.026 bAB	0.852 ± 0.053 aA	0.8 ± 0.033 abAB
W1N2	0.704 ± 0.036 bB	0.883 ± 0.025 cB	0.649 ± 0.032 bB	0.831 ± 0.031 aA	0.721 ± 0.044 cC
W1N3	0.782 ± 0.031 bA	0.972 ± 0.053 bA	0.681 ± 0.027 bAB	0.86 ± 0.042 bA	0.767 ± 0.037 cBC
W2N0	0.819 ± 0.047 bAB	1.073 ± 0.022 bA	0.766 ± 0.029 bcA	0.943 ± 0.042 abA	0.88 ± 0.042 aA
W2N1	0.765 ± 0.032 bAB	1.021 ± 0.05 aAB	0.718 ± 0.036 bA	0.909 ± 0.036 aA	0.831 ± 0.047 abAB
W2N2	0.747 ± 0.034 bB	0.914 ± 0.02 bcC	0.659 ± 0.023 bB	0.881 ± 0.054 aA	0.767 ± 0.032 bcB
W2N3	0.834 ± 0.039 abA	0.985 ± 0.049 bBC	0.77 ± 0.033 aA	0.913 ± 0.043 abA	0.791 ± 0.028 bcB
W3N0	0.983 ± 0.038 aA	1.243 ± 0.044 aA	0.848 ± 0.038 aA	1.014 ± 0.062 aA	0.926 ± 0.055 aA
W3N1	0.891 ± 0.037 aB	1.064 ± 0.028 aBC	0.801 ± 0.042 aAB	0.938 ± 0.037 aAB	0.899 ± 0.034 aA
W3N2	0.852 ± 0.045 aB	1.022 ± 0.055 aC	0.772 ± 0.034 aB	0.909 ± 0.035 aB	0.852 ± 0.033 aA
W3N3	0.903 ± 0.037 aB	1.137 ± 0.029 aB	0.808 ± 0.031 aAB	0.951 ± 0.037 aAB	0.866 ± 0.043 aA
ANOVA					
W	**	**	**	**	**
N	**	**	**	**	**
W × N	ns	ns	ns	ns	ns

Note: W indicates water regulation, N indicates nitrogen fertilizer regulation, W × N indicates the interaction between the two, ns indicates no significant difference (*p* > 0.05), and ** indicates a highly significant difference (*p* < 0.01). Lowercase letters indicate differences between the four irrigation levels at the same nitrogen application rate, while uppercase letters indicate differences between the four nitrogen application rates at the same irrigation level.

**Table 3:** Nitrogen translocation in alfalfa under different water and nitrogen regulations.

Treatment	Transshipment Volume in 2021/(kg·hm^−2^)		Transshipment Volume in 2022/(kg·hm^−2^)		
	1st Harvest	2nd Harvest	1st Harvest	2nd Harvest	1st Harvest
W0N0	34.6 ± 2.26 abA	28.2 ± 2.25 abB	60.7 ± 6.53 aB	50.6 ± 5.07 aB	35.9 ± 2.50 abB
W0N1	37.4 ± 1.36 abA	32.7 ± 2.07 abA	72.1 ± 6.70 aAB	58.6 ± 5.65 aAB	43.7 ± 3.31 aA
W0N2	38.1 ± 3.19 aA	33.7 ± 2.19 abA	75.4 ± 7.41 abA	63.8 ± 5.89 aA	46.3 ± 3.52 aA
W0N3	36.5 ± 1.36 aA	31.9 ± 2.07 abAB	70.5 ± 6.74 aAB	56.5 ± 5.68 aAB	40.4 ± 2.88 abAB
W1N0	36.8 ± 2.22 aA	31.7 ± 2.24 aA	62.4 ± 6.45 aB	49.5 ± 4.39 aB	37.8 ± 2.40 aB
W1N1	39.1 ± 3.09 aA	35.3 ± 3.21 aA	75.1 ± 6.90 aAB	63.1 ± 5.82 aA	45.8 ± 3.16 aA
W1N2	40.0 ± 3.38 aA	35.5 ± 3.47 aA	80.9 ± 7.82 aA	66.1 ± 5.76 aA	48.8 ± 3.08 aA
W1N3	38.2 ± 2.57 aA	33.9 ± 3.31 aA	69.9 ± 7.13 aAB	58.1 ± 6.01 aAB	44.4 ± 2.75 aA
W2N0	33.9 ± 2.15 abA	26.1 ± 1.77 bcB	55.7 ± 5.21 abB	45.1 ± 4.17 aC	33.3 ± 1.94 bB
W2N1	35.2 ± 2.62 abA	29.5 ± 1.79 bAB	69.4 ± 6.97 aA	56.0 ± 5.88 aAB	35.0 ± 1.94 bAB
W2N2	35.9 ± 4.13 aA	30.4 ± 1.88 bcA	72.0 ± 6.47 abA	59.3 ± 5.46 aA	38.8 ± 3.24 bA
W2N3	35.4 ± 3.13 aA	28.5 ± 2.07 bcAB	63.5 ± 6.78 bcAB	49.0 ± 4.72 aBC	35.8 ± 2.21 bcAB
W3N0	31.6 ± 1.83 bA	23.0 ± 2.17 cA	49.1 ± 4.76 bB	42.5 ± 3.68 aB	28.9 ± 2.15 cB
W3N1	33.9 ± 1.63 bA	25.0 ± 2.13 cA	61.9 ± 7.17 aA	52.9 ± 6.05 aA	29.7 ± 2.39 cB
W3N2	35.5 ± 2.91 aA	25.8 ± 1.98 cA	62.8 ± 6.97 bA	56.9 ± 5.88 aA	34.9 ± 2.25 bA
W3N3	33.5 ± 2.05 aA	24.7 ± 2.21 cA	56.1 ± 5.08 cAB	50.1 ± 5.13 aAB	33.1 ± 2.54 cAB
ANOVA					
W	**	*	*	**	**
N	*	*	**	**	**
W × N	*	**	*	**	**

Note: W indicates water regulation, N indicates nitrogen fertilizer regulation, W × N indicates the interaction between the two, * indicates a significant difference (*p* < 0.05), and ** indicates a highly significant difference (*p* < 0.01). Lowercase letters indicate differences between the four irrigation levels at the same nitrogen application rate, while uppercase letters indicate differences between the four nitrogen application rates at the same irrigation level.

**Table 4:** Changes in soil nitrogen content under different treatments.

Treatment	Nitrogen Application Rate (kg·hm^−2^)	Alfalfa Nitrogen Fixation (kg·hm^−2^)	Total Nitrogen Input (kg·hm^−2^)	Gas Losses (kg·hm^−2^)	Nitrogen Uptake by Alfalfa (kg·hm^−2^)	Nitrogen Uptake of Goji Berries (kg·hm^−2^)	Soil Nitrogen Change (kg·hm^−2^)
W0N0	0	200	200	40	80	100	−20
W0N1	150	200	350	70	90	150	40
W0N2	300	200	500	100	100	250	50
W0N3	450	200	650	130	100	350	70
W1N0	0	200	200	30	80	100	−10
W1N1	150	200	350	52.5	90	150	57.5
W1N2	300	200	500	75	100	250	75
W1N3	450	200	650	97.5	100	350	102.5
W2N0	0	200	200	20	80	100	0
W2N1	150	200	350	35	90	150	75
W2N2	300	200	500	50	100	250	100
W2N3	450	200	650	65	100	350	135
W3N0	0	200	200	10	80	100	10
W3N1	150	200	350	17.5	90	150	92.5
W3N2	300	200	500	25	100	250	125
W3N3	450	200	650	32.5	100	350	167.5

**Table 5:** Weights of various indicators calculated based on the entropy weight method.

Indicator	Information Entropy	Information Utility Valued	Weighting (%)
SLR	0.951	0.049	9.6
RW	0.913	0.087	17.2
RSR	0.938	0.062	12.2
NAC	0.936	0.064	12.6
NLCR	0.947	0.053	10.4
NT	0.943	0.057	11.3
HY	0.939	0.061	12.0
NPE	0.925	0.075	14.7

Note: SLR (Stem to Leaf Ratio), RW (Root Weight), RSR (Root to Shoot Ratio), NAC (Nitrogen Accumulation), NLCR (Nitrogen Leaf Contribution Rate), NT (Nitrogen Translocation), HY (Hay Yield), NPE (Nitrogen Production Efficiency).

**Table 6:** Basic physical and chemical properties of soil.

Bulk Density (g·cm^−3^)	Organic Matter Content (g·kg^−1^)	Total N Content (g·kg^−1^)	Total P Content (g·kg^−1^)	Total K Content (g·kg^−1^)	Available N Content (mg·kg^−1^)	Available P Content (mg·kg^−1^)	Available K Content (mg·kg^−1^)	Field Capacity (%)	pH
1.63	6.09	1.62	1.32	34.0	74.5	26.3	173	24.1%	8.11

**Table 7:** Sampling dates for different fertility stages of alfalfa.

Year	Harvest	Moving Date		
		Branching Stage	Budding Stage	Initial Flowering Stage
2021	1st harvest	06-13	07-04	07-20
	2nd harvest	08-19	09-07	09-19
2022	1st harvest	04-26	05-15	05-28
	2nd harvest	06-17	07-07	07-15
	3rd harvest	08-15	09-06	09-20

**Table 8:** Experimental treatments and irrigation water volumes.

Treatment	Nitrogen Application Rate (kg·hm^−2^)	Irrigation Lever	The Lower Irrigation Limits	The Higher Irrigation Limits	Irrigation Water Volume (mm)	
					2021	2022
W0N0	0	Full irrigation	75% θ_fc_	85% θ_fc_	393	471
W0N1	150				403	491
W0N2	300				402	482
W0N3	450				404	481
W1N0	0	Mild water deficit	65% θ_fc_	75% θ_fc_	333	383
W1N1	150				341	408
W1N2	300				307	381
W1N3	450				314	367
W2N0	0	Moderate water deficit	55% θ_fc_	65% θ_fc_	282	327
W2N1	150				287	346
W2N2	300				293	344
W2N3	450				260	304
W3N0	0	Severe water deficit	45% θ_fc_	55% θ_fc_	223	262
W3N1	150				194	254
W3N2	300				217	264
W3N3	450				184	262

## Data Availability

All data are included in this article.

## References

[1] Mo X.G., Liu W., Meng C.C., Hu S., Liu S.X., Lin Z.H. (2021). Variations of forage yield and forage-livestock balance in grass-lands over the Tibetan Plateau, China. J. Appl. Ecol..

[2] Ma J.L., Cheng W.Y. (2023). The Feeding Value and High-yield Cultivation Technology of Alfalfa. Tillage Cultiv..

[3] Silitonga A.S., Riayatsyah T.M., Kalam M.A., Sarifudin A., Fattah I.M., Muraza O., Putra N.S., Sebayang A.R., Sebayang A.H., Hermawan H. (2025). Status, developments, and sustainability of biowaste feedstock: A review of current progress. Renew. Sustain. Energy Rev..

[4] Danishta A., Summira R., Pawan S., Ishtiyaq A., Basanagouda G., Sheikh A.R., Shafiya R., Pooja S., Gulab K.R., Khursheed A. (2025). Remote sensing and artificial intelligence: Revolutionizing pest management in agriculture. Front. Sustain. Food Syst..

[5] Mahima C., Sarita K., Prem K., Muthukumaran P., Bandana K.S., Kamaljit K., Parul S., Raman K., Abishek K., Vijayakumar S. (2025). Rational fertilizer application dream: Ammonia gas sensor for monitoring urea loss from crops. Microchem. J..

[6] Li S., Wang S.J., Shi J.L., Tian X.H., Ye X.X. (2023). Integrated mulching and nitrogen management strategies influence carbon footprint and sustainability of wheat production on the Loess Plateau of China. Field Crops Res..

[7] Dang P.F., Lu C., Huang T.T., Zhang M.M., Yang N., Han X.Q., Xu C.H., Wang S.G., Wan C.X., Qin X.L. (2024). Enhancing intercropping sustainability: Manipulating soybean rhizosphere microbiome through cropping patterns. Sci. Total Environ..

[8] Poloko M., Moeketsi N., Tumelo N., Palo L. (2025). The Role of Intercropping Selected Maize Cultivars and Forage Legumes on Yield, Weed Dynamics, and Soil Chemical Properties. Int. J. Agron..

[9] Agnese B., Gergely U., Matteo D., Filippo R., Claudia B., Roberta P., Giacomo B., Carlo V. (2025). The impact of alfalfa inter-cropping and conventional tillage on N-cycling microbes: A Tuscan vineyard case study. Appl. Soil Ecol..

[10] Feng X.J., Zhong M.H., Zhao X.X., Zhang X.L., Hu Y.B., Zhang H.H. (2025). Intercropping Forage Mulberry Benefits Nodulation and Growth of Soybeans. Agronomy.

[11] Li M.Z., Wei Y.W., Yin Y., Zhu W.X., Bai X.J., Zhou Y.B. (2023). Characteristics of Soil Physicochemical Properties and Microbial Community of Mulberry (*Morus alba* L.) and Alfalfa (*Medicago sativa* L.) Intercropping System in Northwest Liaoning. Microorganisms.

[12] Zhang G.G., Dong S.T., Yang Z.B. (2011). Production performance of alfalfa + maize intercropping systems and evaluation of interspecies competition. Acta Pratacult. Sin..

[13] Ma Y., Cao B., Wang X.J., Feng L.X. (2025). Intercropping ryegrass with ‘LingwuChangzao’ (Ziziphus jujuba Mill. cv. Lingwu-Changzao) enhances crop yield and quality in the arid regions of Northern China. Agroforest. Syst..

[14] Yang J.J., Finn D.R., Wang H.T., Joachim B., Tebbe C.C. (2025). Seasonal dynamics of prokaryotic nitrogen cycling genes in cropland soils: Effects of soil texture, tillage, and environmental factors. Soil Tillage Res..

[15] Wang D.L., Liu S.B., Guo M.J., Cheng Y.H., Shi L.F., Li J.P., Yu Y.J., Wu S.Y., Dong Q.G., Ge J.K. (2025). Optimizing Nitrogen Fertilization and Irrigation Practices for Enhanced Winter Wheat Productivity in the North China Plain: A Meta-Analysis. Plants.

[16] Kang Y.X., Qi G.P., Jia Q., Wang A.X., Yin M.H., Ma Y.L., Wang J.H., Jiang Y.B., Tang Z.X. (2023). Appropriate Water-Nitrogen Regulation Mode to Improve Productivity of Mixed-Sowing Grassland of *Bromus inermis* and Alfalfa. Water.

[17] Ma H.X., Sun Q., Zhang X.J., Jiang P. (2025). Effects of subsurface drip irrigation and nitrogen fertilizer management on N_2_O emissions and forage yield in alfalfa production. Front. Plant Sci..

[18] Yin M.H., Jiang Y.B., Ling Y., Ma Y.L., Qi G.P., Kang Y.X., Wang Y.Y., Lu Q., Shang Y.J., Fan X.R. (2025). Optimizing Lucerne Productivity and Resource Efficiency in China’s Yellow River Irrigated Region: Synergistic Effects of Ridge-Film Mulching and Controlled-Release Nitrogen Fertilization. Agriculture.

[19] Yang T., Lu W.H., Li B., Duan Z.P., Shen L., Teng Y.X., Liu T.T., Tian Y.Q., Zhang W., Li L.H. (2020). Root distribution characteristics and productivity in a poplar-alfalfa silvopastoral system. Agric. Res. Arid Areas.

[20] Zunker S.M.J.H., Knappe H. (2024). (De)politicizing water: Justice in times of water crisis. Front. Polit. Sci..

[21] Wang C., Qi G.P., Ma Y.L., Yin M.H., Wang J.H., Kang Y.X., Jia Q., Gao Y.L., Tian R.R., Zhang R. (2024). Effects of Water and Nitrogen Control on the Growth Physiology, Yields, and Economic Benefits of Lycium barbarum Plants in a Lycium barbarum + Alfalfa System. Plants.

[22] Liang X., Li Y.S., Li W.H., Yuan G.D., Wu J.H., Han F.X., Chen M.H. (2025). Co-applications of Biochar and Reduced Fertilizer Improved Soil Fertility, Nitrogen Use Efficiency, and Yield of Lycium chinense Mill: A Two-Year Field Trial. J. Soil Sci. Plant Nutr..

[23] Wang Z.Y., Shang J.X., Wang X.L., Ye R.Q., Zhao D., Li X.Y., Yang Y.D., Zhang H.Y., Gong X.W., Jiang Y. (2024). Soil Greenhouse Gas Emissions and Nitrogen Dynamics: Effects of Maize Straw Incorporation Under Contrasting Nitrogen Fertilization Levels. Agronomy.

[24] Graham S.L., Laubach J., Hunt J.E., Mudge P.L., Nuñez J., Rogers G.N.D., Buxton R.P., Carrick S., Whitehead D. (2022). Irrigation and grazing management affect leaching losses and soil nitrogen balance of Lucerne. Agric. Water Manag..

[25] Wang Y.D., Zhang Q.C., Ju M.X., Gao K., Han L.L., Li X.F., He J., Su D.R. (2025). Alfalfa Photosynthesis Under Partial Root-Zone Drying: Diurnal Patterns and Its Non-Stomatal Limitations. Plants.

[26] Crookston S.B., Boren D., Yost M., Sullivan T., Creech E., Barker B., Reid C. (2025). Irrigation technology, irrigation dose, and crop genetic impacts on alfalfa yield and quality. Agric. Water Manag..

[27] Wang Y.D., Zhang Q.C., Gao K., Han L.L., Li X.F., He J., Su D.R. (2025). Deficit Irrigation Provides a Trade-Off Between Water Use and Alfalfa Quality. Agronomy.

[28] Liu J., Qi G.P., Kang Y.X., Ma Y.L., Yin M.H., Li X.M., Li Z. (2019). Photosynthetic Characteristics, Chlorophyll Fluorescence Parameters and Biomass of Alfalfa under Different Irrigation Treatments. Acta Agrestia Sin..

[29] Li Q.Q., Bian C.Y., Liu X.H., Ma C.J., Liu Q.R. (2015). Winter wheat grain yield and water use efficiency in wide-precision planting pattern under deficit irrigation in North China Plain. Agric. Water Manag..

[30] Liu L., Li P.W., Xu M.G., Ma H.Y., Yang X.Y., Zhang S.L. (2025). Effects of soil fertility and N application rates on soil organic N components and N mineralization. J. Agro-Environ. Sci..

[31] Sha B.P., Xie Y.Z., Gao X.Q., Cai W., Fu B.Z. (2021). Effects of coupling of drip irrigation water and fertilizer on yield and quality of alfalfa in the yellow river irrigation district. Acta Pratacult. Sin..

[32] Liu C., Wang Y.D., Cui P.F., Su D.R. (2021). Effects of Different Irrigation Limits on Growth and Photosynthetic Characteristics of Alfalfa in Arid Region of Northwest China. Chin. J. Grassl..

[33] Ren H.P., Ning S.R., Yan A., Zhao Y.Q., Li N., Huo T.T. (2025). Response of Alfalfa Yield to Rates and Ratios of N, P, and K Fertilizer in Arid and Semi-Arid Regions of China Based on Meta-Analysis. Agronomy.

[34] Liu M.G., Wang Z.K., Mu L., Xu R., Yang H.M. (2021). Effect of regulated deficit irrigation on alfalfa performance under two irrigation systems in the inland arid area of midwestern China. Agric. Water Manag..

[35] Mu G.Y., Jiang Y.B., Li H.Y., Wei S.N., Qi G.P., Kang Y.X., Yin M.H., Ma Y.L., Wang Y.Y., Wang Y.B. (2025). Water–Fertilizer Synergistic Effects and Resource Optimization for Alfalfa Production: A Central Composite Design and Response Surface Methodology Approach. Plants.

[36] Liu B., Brooks E., Mohamed A.Z., Kelley J. (2024). Deep Infiltration Model to Quantify Water Use Efficiency of Center-Pivot Irrigated Alfalfa. J. Irrig. Drain. Eng..

[37] Elgharably A., Benes S. (2021). Alfalfa Biomass Yield and Nitrogen Fixation in Response to Applied Mineral Nitrogen Under Saline Soil Conditions. J. Soil Sci. Plant Nutr..

[38] Chen Y.Q., Liu J.Y., Liu W.K. (2023). Enhancing growth, quality, and metabolism of nitrogen of alfalfa (*Medicago sativa* L.) by high red–blue light intensity. J. Plant Nutr. Soil Sci..

[39] Bai Z.H., Lu J., Zhao H., Velthof G.L., Oenema O., Chadwick D., Williams J.R., Jin S.Q., Liu H.B., Wang M.R. (2018). Designing Vulnerable Zones of Nitrogen and Phosphorus Transfers To Control Water Pollution in China. Environ. Sci. Technol..

[40] Miao F.H., Yu X.X., Tang X.K., Liu X.D., Tang W., Zhao Y.H., Yang C., Xu Y.F., Yang G.F., Sun J. (2023). The Responses of Stem and Leaf Functional Traits of *Medicago sativa* and *Bromus inermis* to Different Mixed Planting Patterns. Agronomy.

[41] Wang Y.D., Liu C., Cui P.F., Su D.R. (2020). Effects of partial root-zone drying on alfalfa growth, yield and quality under sub-surface drip irrigation. Agric. Water Manag..

[42] Zhang L.Y., Zhang J.R., Liu F., Yao B. (2014). A review of ecological benefits of silvopasture systems. Pratacult. Sci..

[43] Ma B., Teng Y., Wang X., Lv W., Wu X.Z., Wang Z.T. (2025). Response of soil moisture to rainfall in alfalfa fields under severe soil drying condition. Acta Ecol. Sin..

[44] Imène B.S., Alfonso A., Cristina M.A., Rabiaa H., Nehla L., Fethia Z., Vicente M., Francisco P.A., Chedly A. (2008). Response of nitrogen fixation in relation to nodule carbohydrate metabolism in Medicago ciliaris lines subjected to salt stress. J. Plant Physiol..

[45] Shao Z.Q., Zheng C.C., Postma J.A., Gao Q., Zhang J.J. (2024). More N fertilizer, more maize, and less alfalfa: Maize benefits from its higher N uptake per unit root length. Front. Plant Sci..

[46] Karan T., Moran B. (2025). Alfalfa (Medicago sativa) Leaves Mediated Synthesis of Silver Nanoparticles and Assessment of Their Cytotoxic Effects on Various Cancer Cell Lines. Biol. Bull..

[47] Guo L.F., Zhang X.C., Liu Y.N., Zhang A.Q., Song W.S., Li L.X., Zhao J.W., Pang Q.Y. (2025). Salt-alkali-tolerant growth-promoting Streptomyces sp. Jrh8-9 enhances alfalfa growth and resilience under saline-alkali stress through integrated modulation of photosynthesis, antioxidant defense, and hormone signaling. Microbiol. Res..

[48] Lu Q., Qi G.P., Yin M.H., Kang Y.X., Ma Y.L., Jia Q., Wang J.H., Jiang Y.B., Wang C., Gao Y.L. (2024). Alfalfa Cultivation Patterns in the Yellow River Irrigation Area on Soil Water and Nitrogen Use Efficiency. Agronomy.

[49] Su K.Q., Mu L., Zhou T., Kamran M., Yang H.M. (2022). Intercropped alfalfa and spring wheat reduces soil alkali-salinity in the arid area of northwestern China. Plant Soil.

[50] Aurélie Q., Patricia B., Lydie D., Jacques W., Christian D. (2017). Effects of walnut trees on biological nitrogen fixation and yield of intercropped alfalfa in a Mediterranean agroforestry system. Eur. J. Agron..

[51] Zhang M.M., Wang N., Hu Y.B., Sun G.Y. (2018). Changes in soil physicochemical properties and soil bacterial community in mulberry (*Morus alba* L.)/alfalfa (*Medicago sativa* L.) intercropping system. Microbiol. Open.

[52] Lv Y.R., Wang J.H., Yin M.H., Kang Y.X., Ma Y.L., Jia Q., Qi G.P., Jiang Y.B., Lu Q., Chen X.L. (2023). Effects of Planting and Nitrogen Application Patterns on Alfalfa Yield, Quality, Water–Nitrogen Use Efficiency, and Economic Benefits in the Yellow River Irrigation Region of Gansu Province, China. Water.

[53] Liu H.F., Nan Z.B., Tang Z., Wang L.J. (2016). Comparison of economic benefit of alfalfa, wheat and maize—A case study in Gansu Province. Pratacult. Sci..

[54] Yan S.D., Jiang H.S., Dong X.Y., Zhang Y.H., Guo T.W., Han Y., Liu Y., Yan Q.Y. (2024). Effects of Reduced Application of Slow/Controlled Release Ammonium Sulfate on Maize Growth and Soil Nitrogen Balance. J. Nucl. Agric. Sci..

